# Mechanical and Microstructural Behavior of Drinking Water Treatment Sludge Stabilized with Eggshell-Derived Hydrated Lime and Commercial Lime

**DOI:** 10.3390/ma19132692

**Published:** 2026-06-23

**Authors:** Camilo Andrés Cabarcas Castro, Camilo Andrés Angulo Batista, Luis Carlos Suárez López, Yamid E. Nuñez de la Rosa, Manuel Saba, Monica Eljaiek-Urzola, Jair Arrieta Baldovino

**Affiliations:** 1 Civil Engineering Program, Universidad de Cartagena, Cartagena de Indias 130015, Colombia; ccabarcasc@unicartagena.edu.co (C.A.C.C.); cangulob1@unicartagena.edu.co (C.A.A.B.); lsuarezl@unicartagena.edu.co (L.C.S.L.); msaba@unicartagena.edu.co (M.S.); meljaieku@unicartagena.edu.co (M.E.-U.); 2 Faculty of Engineering and Basic Sciences, Fundación Universitaria Los Libertadores, Bogota 110231, Colombia

**Keywords:** drinking water treatment sludge, eggshell lime, alkali activation, strength, soil stabilization

## Abstract

The valorization of drinking water treatment sludge (DWTS) and eggshell waste represents a promising route for reducing landfill disposal and developing alternative stabilized materials for geotechnical applications. This study aimed to evaluate the mechanical and microstructural behavior of DWTS stabilized with commercial lime (CL) and eggshell-derived hydrated lime (EHL), including alkali-activated EHL systems. EHL was produced from locally collected eggshell waste through washing, drying, grinding, calcination at 1000 °C for 4 h, hydration, drying, and sieving. The mixtures were prepared with lime contents of 5%, 8%, 11%, and 14%, while NaOH solutions of 0.5, 1.0, and 1.5 M were used for the activated systems. A total of 120 cylindrical specimens were compacted under controlled dry unit weight and moisture content and cured for 7 and 28 days. The stabilized DWTS was evaluated through unconfined compressive strength (q_u_), SEM–EDS analysis, and multifactorial ANOVA. The highest q_u_ for CL-treated specimens was 4561.72 kPa at 14% lime and 28 days, while EHL reached its best response at 11% lime and 7 days, with a q_u_ of 3195.13 kPa. In general, EHL showed a competitive performance at intermediate and high lime contents, although increasing NaOH molarity tended to reduce strength.

## 1. Introduction

The increasing demand for building materials and the depletion of natural resources have prompted the search for sustainable alternatives to conventional raw materials. In this context, drinking water treatment sludge (DWTS) has attracted attention as an abundant solid waste that is difficult to manage and may raise environmental concerns if not properly valorized [1]. The use of aluminum-based coagulants in drinking water treatment also generates large amounts of Al-rich sludge, which is commonly sent to landfills, occupying valuable urban land and increasing disposal-related risks [2].

Different types of industrial and municipal sludge have been studied as alternative materials for construction applications, including bricks, lightweight products, concrete paving blocks, cementitious composites, and geopolymer-based systems [3,4,5]. In the case of DWTS, its use has also been explored from a geotechnical perspective, either as a soil additive or as part of stabilized systems for earthworks, pavement layers, and subgrade improvement [6]. Recent studies have shown that DWTS can improve some engineering properties when combined with stabilizers such as lime, cement, fly ash, or other additives. However, its response depends strongly on the mixture design, curing conditions, and binder type [7,8,9].

The interest in DWTS is also related to its aluminosilicate nature, which may enable its use in cement replacement, alkali-activated binders, and geopolymer systems [10,11]. However, its direct use is not always straightforward, as high sludge content may increase porosity, water absorption, and shrinkage, or reduce mechanical performance if the material is not properly treated or combined with a suitable stabilizing agent [4,5,10,12]. Therefore, calcium-rich binders remain relevant for improving the mechanical response of DWTS-based mixtures, opening the possibility of using not only commercial lime but also alternative calcium sources such as eggshell-derived lime.

The use of materials derived from eggshell waste, particularly eggshell powder and eggshell-based lime, has emerged as a sustainable alternative to conventional stabilizers such as cement and lime. This growing interest is primarily driven by the high calcium carbonate content of eggshells (95% of CaCO_3_), which provides significant potential as a cementitious agent or precursor in soil stabilization processes. Several studies have demonstrated that these materials can substantially improve the mechanical and durability properties of soils while simultaneously reducing the environmental impact of construction activities. Lu and Hu [13] investigated the stabilization of expansive soils using a composite of eggshell ash and silica fume, emphasizing the importance of synergistic interactions between calcium-rich and silica-rich materials. Their findings showed that a mixture containing 9% eggshell and 8% silica fume reduced the free swell ratio to below 20% and increased the unconfined compressive strength (q_u_) to 1.90 MPa after 28 days of curing. This behavior was attributed to the formation of cementitious products, primarily calcium silicate hydrate (C–S–H) gels, as well as to the compression of the diffuse double layer induced by Ca^2+^ ions. Similarly, Ardejani et al. [14] investigated the partial replacement of lime with eggshell powder for the stabilization of kaolinitic clay, demonstrating that incorporating 25% eggshell powder as a lime substitute significantly enhances both the mechanical performance and durability of the soil. The authors reported a more than fourfold increase in q_u_ and a sixfold improvement in indirect tensile strength when the system was combined with hemp fibers. Microstructural analyses confirmed the formation of C–S–H and C–A–H gels, indicating the active participation of eggshell powder in pozzolanic reactions under appropriate conditions.

The effectiveness of eggshell has also been validated in systems incorporating biopolymers. Kalita et al. [15] examined the stabilization of silty sand using xanthan gum and eggshell, reporting that a mixture containing 1% biopolymer and 6% ESP increased the California Bearing Ratio (CBR) by more than 300%. This improvement was attributed to the development of a denser and more cohesive matrix, as evidenced by scanning electron microscopy. In comparative studies, Nierwinski et al. [16] demonstrated that eggshell-derived lime exhibits superior mechanical performance compared to commercial lime under similar experimental conditions. Complementarily, Srirama and Jayanthi identified that an optimal eggshell lime content of approximately 7% results in significant improvements in q_u_, CBR, cohesion, and the internal friction angle, consistently outperforming conventional hydrated lime.

The use of eggshell powder as an additive in cement-stabilized systems has also been widely reported. Alqaisi et al. [17] demonstrated that the addition of 6% eggshell powder to a soil treated with 6% cement significantly improves shear strength and CBR, while reducing swelling potential. Likewise, Hasriana et al. [18] report an up to 11-fold increase in CBR when 5% eggshell powder was incorporated into soils stabilized with 5% cement, highlighting the strong synergistic interaction between the two materials. In clayey soils, Chen et al. [19] analyzed the combined use of lime and eggshell powder. They found that the optimal eggshell powder content ranges from 10% to 15%, as higher dosages reduce mechanical properties. This behavior suggests that excessive eggshell powder may result in unreacted particles, thereby reducing the efficiency of the stabilization process. In contrast, moderate content promotes flocculation, cementation, and pore filling, leading to significant gains in strength.

The incorporation of eggshell powder in combination with fibers has also shown promising results. Eshghi et al. [20] reported that a mixture containing 10% eggshell powder and 1% polypropylene fibers improves compressive strength, stiffness, and ultrasonic pulse velocity in clayey soils. The inclusion of fibers further enhances ductility and energy absorption capacity.

In more advanced systems, Tanyıldızı [21] examined the application of eggshell powder in combination with alkali-activated materials, demonstrating significant improvements in strength, reduction in swelling potential, and lower water absorption. Additionally, the study reported an approximate 30% reduction in CO_2_ emissions compared to Portland cement-based systems, reinforcing the sustainability of these approaches. Other studies have explored the application of ESP in marginal soils. Lacheheub et al. [22] demonstrated that the combination of eggshell lime and natural zeolite increases the strength of marl soils by up to eleven times. Similarly, Yang et al. [23] reported increases of up to 1000% in q_u_ and 420% in CBR using calcined eggshell powder, with optimal contents ranging between 6% and 8%. Despite these advances, several important limitations remain. The effectiveness of eggshell powder strongly depends on its dosage, particle size, and degree of thermal activation. In general, optimal content ranges from 4% to 10%, although it may vary depending on the stabilizing system and soil type. Moreover, eggshell powder alone exhibits limited reactivity compared to conventional stabilizers, and therefore, its application typically requires a combination with pozzolanic materials, cement, or alkali-activated systems.

Although previous studies have demonstrated the potential of DWTS stabilization with conventional binders and eggshell-derived materials as alternative calcium sources, several important knowledge gaps remain. First, limited information is available regarding the stabilization of DWTS using eggshell-derived hydrated lime (EHL) as a direct substitute for commercial lime. Second, the influence of alkaline activation on EHL-stabilized DWTS has not been systematically investigated, particularly in systems containing predominantly crystalline silica phases. Third, few studies have linked mechanical performance, statistical evaluation, and microstructural evolution to explain the effectiveness of alternative lime sources in DWTS stabilization. Consequently, the mechanisms governing the interaction between EHL, alkaline activators, and DWTS remain poorly understood.

Thus, this study evaluates the mechanical and microstructural behavior of DWTS stabilized with CL and EHL, including EHL mixtures activated with NaOH. The work compares non-activated and alkali-activated systems to assess the feasibility of using EHL as an alternative calcium-based stabilizer. For this purpose, EHL was produced from locally collected eggshell waste through washing, drying, grinding, calcination, hydration, and sieving. The mixtures were prepared with lime contents of 5%, 8%, 11%, and 14%, while NaOH concentrations of 0.5, 1.0, and 1.5 M were used for the activated EHL systems. All specimens were compacted under the same dry unit weight and moisture content and cured for 7 and 28 days. The q_u_ tests, SEM–EDS observations, and multifactorial ANOVA were then used to evaluate the effect of lime source, EHL content, NaOH concentration, curing time, and their interactions on the stabilized DWTS response.

## 2. Materials and Methods

**Figure 1:**
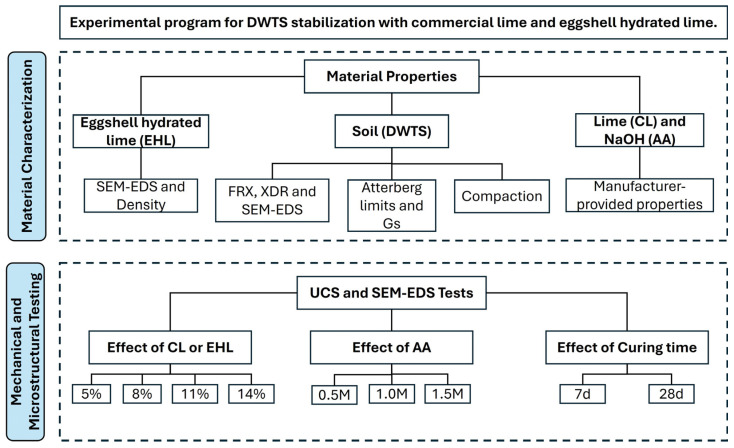
Experimental program for DWTS stabilization.

The experimental program focused on stabilizing DWTS using EHL and CL. Considering that the processing conditions and stabilization potential of eggshell-derived lime have been previously studied with favorable results [15,16], this work was organized into two main stages (Figure 1). The first stage involved the physical, chemical, and mineralogical characterization of the raw materials, with special emphasis on the DWTS. The second stage evaluated the mechanical and microstructural response of the stabilized specimens, considering the effects of binder type, binder content, alkaline activation, and curing time on q_u_ and SEM–EDS observations.

### 2.1. Materials

#### 2.1.1. Drinking Water Treatment Sludge

The DWTS used in this study was collected from the El Bosque drinking water treatment plant in Cartagena de Indias, Colombia, located at 10°23′56.54″ N and 75°30′44.91″ W. After collection, the sludge was stored in plastic containers and transported to the laboratory. The material was then dried in small batches at 100 °C for approximately 24 h to reduce its moisture content. Due to the formation of hardened lumps during drying, the dried DWTS was manually crushed to obtain a more workable material and then stored in sealed containers until further use. The main physical and chemical properties of the DWTS are summarized in Table 1.

**Figure 2:**
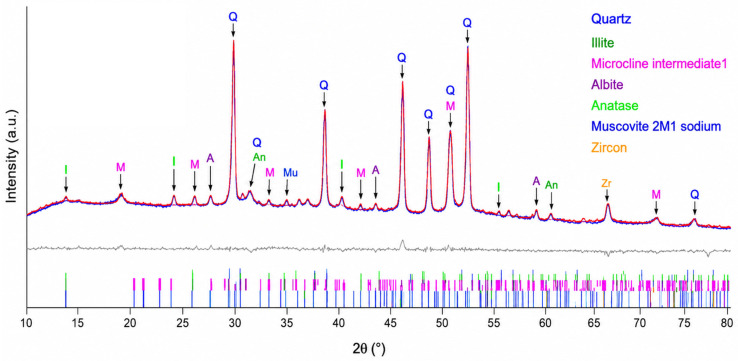
Mineralogical composition of the DWTS sample determined by XRD.

The mineralogical composition of the DWTS sample is summarized in Figure 2. The analysis was performed by X-ray diffraction (XRD) on powder mounts using a Bruker D4 Endeavor diffractometer equipped with a LYNXEYE detector (Bruker AXS GmbH, Karlsruhe, Germany), Cu Kα radiation source (λ = 1.5406 Å), and Ni filter. The equipment was operated at 40 kV and 30 mA, with a 2θ scanning range of 5° to 70°, a step size of 0.015°, and a scanning speed of 0.4 s per step. For amorphous phase determination, ZnO was used as an internal standard at a sample-to-standard ratio of 8:2, and the mineral phases were quantified using DIFFRAC.TOPAS Version 6 (Bruker AXS GmbH, Karlsruhe, Germany). based on the Rietveld refinement method. The results show that the DWTS sample is mainly composed of amorphous material, followed by quartz, muscovite, kaolinite, illite, plagioclase, anatase, and K-feldspar.

The chemical composition of the DWTS sample is presented in Table 2, where the major elements are reported as oxides (% *w*/*w*) and the detected trace elements are expressed in parts per million (ppm). The analysis was performed by X-ray fluorescence (XRF) using a Bruker S4 Explorer WDS-XRF spectrometer (Bruker AXS GmbH, Karlsruhe, Germany), equipped with a Rh X-ray tube and operated at 38 kV and 26 mA, the data were processed using Bruker-AXS F-Quant software under ambient laboratory conditions. Only the quantified compounds and elements are included in the final table, while those not detected or below the quantification limit were excluded from the report.

**Figure 3:**
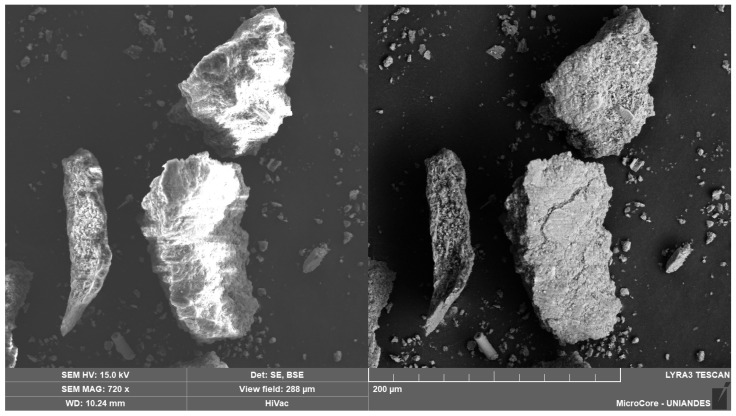
SEM micrograph of the DWTS sample.

Figure 3 shows the SEM micrograph of the DWTS sample. The material is mainly composed of irregular particles with angular and elongated shapes, lacking a defined or uniform morphology. Several fragments exhibit rough surfaces, microcracks, and porous textures, which are typical of sludge-derived solids after drying and handling. Fine particles are also observed around the larger grains, suggesting a heterogeneous particle arrangement.

#### 2.1.2. Eggshell-Derived Lime

Eggshell waste was collected from commercial establishments in Cartagena, Colombia. The shells were washed to remove surface impurities and residual organic matter, then dried at room temperature (approximately 31 °C) on an absorbent surface. After drying, the eggshells were ground in a mechanical mill until a visually uniform particle size was obtained; however, no particle-size distribution analysis was performed at this stage.

**Figure 4:**
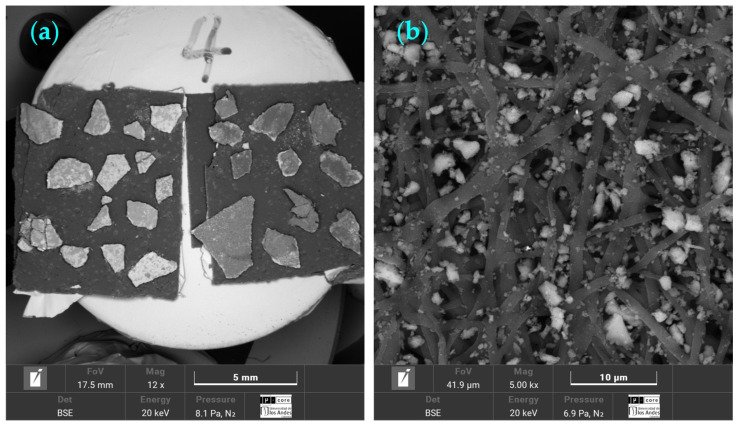
Morphology of the eggshell sample: (**a**) general view at 12× magnification, scale bar = 5 mm; (**b**) surface detail at 5000× magnification, scale bar = 10 µm.

Figure 4 shows the morphology of the crushed eggshells before calcination. The particles exhibit irregular shapes and rough surfaces, characteristic of mechanically ground eggshell waste before thermal treatment.

**Figure 5:**
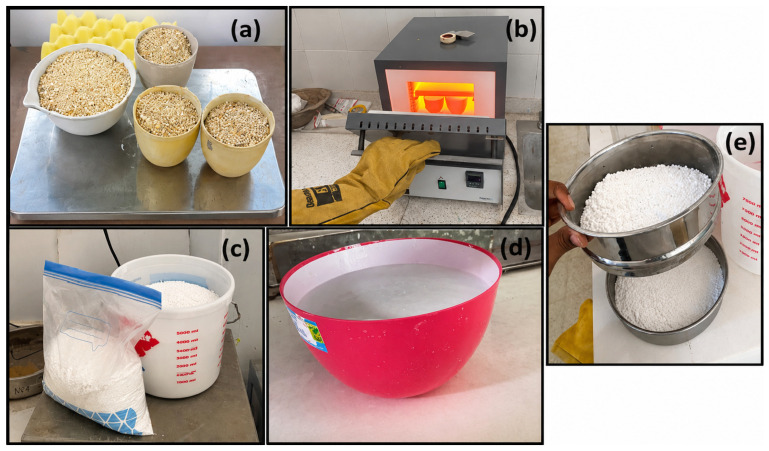
Lime production process: (**a**) Ground eggshells. (**b**) Calcination of eggshells. (**c**) Quicklime. (**d**) Hydration of quicklime. (**e**) Dry and sieved hydrated lime.

The physical properties of the EHL: Fineness was evaluated according to ASTM C977 [26], while particle density was determined following ASTM C110 [27]. The material showed 0% retained on the No. 30 sieve (0.600 mm) and 22% retained on the No. 200 sieve (0.075 mm), complying with the ASTM C977 [26] limits of ≤3% and ≤25%, respectively. Figure 5 presents the process of EHL production. The particle density of EHL was 2.3 g/cm^3^, indicating that the processed material presented suitable physical characteristics for use as a lime-based stabilizing agent.

The ground eggshells were then calcined at 1000 °C for 4 h, excluding the heating ramp, which was applied at 20 °C/min (Figure 5b). Once the eggshell-derived quicklime was obtained, it was hydrated by immersion for 48 h (Figure 5d). The hydrated material was subsequently dried for an additional 48 h at 60 °C, manually disaggregated, and finally passed through a No. 30 sieve with an opening size of 0.600 mm as presented in Figure 5e.

The calcined eggshells were intentionally hydrated before use to produce eggshell-derived hydrated lime (EHL), allowing a direct comparison with the commercial hydrated lime (CL) employed in this study. Although quicklime (CaO) naturally hydrates upon contact with water, controlled hydration was performed to ensure complete conversion of CaO into Ca(OH)_2_ before specimen preparation, thereby minimizing variability associated with incomplete hydration, localized expansion, and heat release during mixing.

**Figure 6:**
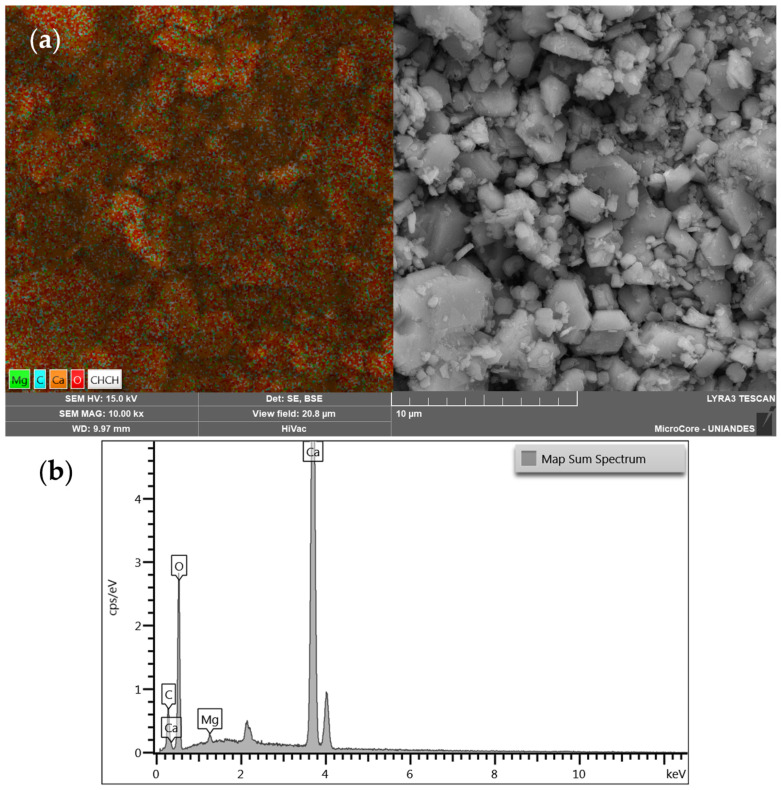
SEM–EDS analysis of the EHL sample: (**a**) SEM micrograph and layered EDS map showing the predominance of calcium in the analyzed area; (**b**) EDS map sum spectrum of the EHL sample.

Figure 6 presents the SEM–EDS analysis of the EHL sample. In Figure 6a, the layered EDS map shows a clear predominance of calcium, which is consistent with the lime-based nature of the material. The semi-quantitative EDS results indicate that the sample is mainly composed of Ca and O, with minor amounts of C and Mg, with Ca at 48.71 wt%, O at 46.53 wt%, C at 4.28 wt%, and Mg at 0.48 wt%. The SEM micrograph also reveals irregular agglomerates and particles with hexagonal-like morphology within a field of view of 20.8 µm, which may be associated with the typical crystalline structure of calcium hydroxide (Ca(OH)_2_). Finally, Figure 6b shows the EDS map sum spectrum, where the most intense calcium peaks are observed between approximately 3.6 and 3.8 keV, reaching values above 4 cps/eV, confirming the strong calcium signal in the analyzed area.

#### 2.1.3. Commercial Lime

The commercial lime (CL) used in this study was hydrated. According to a previous characterization study conducted by Baldovino et al. [28], the material was mainly composed of calcium oxide (CaO) and magnesium oxide (MgO). The hydrated lime presented a specific gravity of 2.39 and a fine particle size distribution, with approximately 91% of the particles passing the No. 200 sieve (0.075 mm). The commercial hydrated lime was adopted as a reference stabilizer to evaluate the feasibility and performance of the eggshell-derived hydrated lime (EHL) produced in this study.

#### 2.1.4. Alkaline Activator

Sodium hydroxide (NaOH) was used as the alkaline activator (AA) in the preparation of the treated mixtures. The reagent was supplied as solid pellets by Merck KGaA under the EMSURE^®^ line (Darmstadt, Germany), with analytical-grade purity. According to the manufacturer’s certificate, the NaOH had a reported purity of 99.6%, a molar mass of 40.00 g/mol, and low levels of carbonate, chloride, and heavy metal impurities [28]. The material was stored in 1 kg sealed containers before use. For the alkaline solutions, the required amount of NaOH was weighed and dissolved in distilled water, then cooled to room temperature before mixing with the lime-treated material, as NaOH dissolution is highly exothermic.

### 2.2. Methodology

#### 2.2.1. Specimen Molding and Preparation

**Figure 7:**
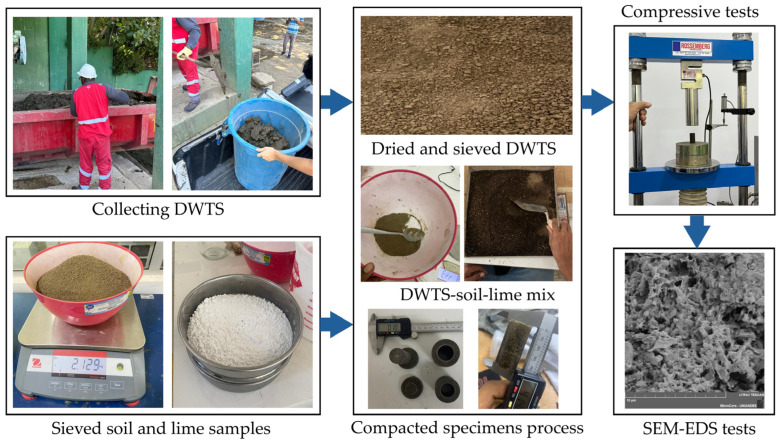
Specimen molding of DWTS-soil-lime mixes and preparation for mechanical and microstructural testing.

The experimental matrix used to prepare the stabilized DWTS specimens is presented in Table 3. The program considered two binder systems: commercial lime (CL) and eggshell hydrated lime (EHL), as well as AA-EHL mixtures prepared with NaOH concentrations of 0.5, 1.0, and 1.5 M. For each binder system, four binder contents (5%, 8%, 11%, and 14%) were evaluated, with curing periods of 7 and 28 days. Each condition was prepared in triplicate, resulting in 24 specimens per binder system and a total of 120 specimens. All samples were molded at a target dry unit weight of 15.6 kN/m^3^ and a moisture content of 29.3%. To ensure uniform specimen preparation, compaction was performed under constant conditions using a 12-ton hydraulic press. Figure 7 presents the specimen molding of DWTS-soil-lime mixes and preparation for mechanical and microstructural testing.

The specimens were prepared following the procedure reported by López et al. [29], maintaining a diameter-to-height ratio of 1:2. Cylindrical samples with a diameter of 21 mm and a height of 42 mm were molded, in accordance with the recommendations of ASTM D1632 [30]. During specimen preparation, strict rejection criteria were applied to ensure dimensional accuracy and density uniformity among the samples [31,32,33]. After molding, the specimens were wrapped in plastic film to minimize moisture loss and then placed in a humid curing chamber at approximately 23 °C and 95% relative humidity until the corresponding curing age. This procedure was adopted to preserve the moisture conditions required for the development of stabilization reactions.

#### 2.2.2. The q_u_ Test

Following recommendations reported in previous studies [29,31], and considering the origin of the DWTS, the specimens were saturated for 24 h before q_u_ testing. This step was included to minimize the contribution of matric suction to the measured strength, allowing the results to better reflect the bonding effect produced by lime stabilization rather than temporary suction-related strength.

The q_u_ tests were performed on cylindrical specimens in accordance with ASTM D2166 [34]. A 50 kN hydraulic press, with a load sensitivity of 0.1 kN, was used for the compression tests. The load was applied at a constant displacement rate of 1.15 mm/min to maintain stable and uniform deformation until specimen failure.

#### 2.2.3. Statistical Analysis

A statistical analysis of the unconfined compressive strength results was performed using analysis of variance (ANOVA) in IBM SPSS Statistics v29. The evaluation used a 5% significance level to examine the effects of the studied variables and their interactions on the mechanical response of the stabilized mixtures. The analyzed factors included stabilizer dosage, NaOH concentration, and curing period. This procedure enabled the identification of statistically significant differences in the average strength values across the evaluated conditions.

#### 2.2.4. Microstructural Analysis

After the q_u_ tests, selected stabilized specimens were prepared for microstructural characterization using a Tescan LYRA-3 SEM-FIB system (TESCAN ORSAY HOLDING, Brno–Kohoutovice, Czech Republic). Before the analysis, the samples were oven-dried at 60 °C for 24 h to reduce residual moisture and preserve the internal structure. Subsequently, representative fragments were mounted on aluminum stubs using conductive carbon tape and, when necessary, coated with a thin gold layer to improve surface conductivity during imaging.

The microstructural observations were carried out at accelerating voltages of 15–20 kV using secondary electron (SE) and backscattered electron (BSE) detectors. In addition, energy-dispersive X-ray spectroscopy (EDS) was employed to determine the elemental composition of selected regions within the stabilized matrices.

## 3. Results and Discussion

### 3.1. Comparison of DWTS Samples Stabilized with CL and EHL Without NaOH at 7 and 28 Days of Curing

**Figure 8:**
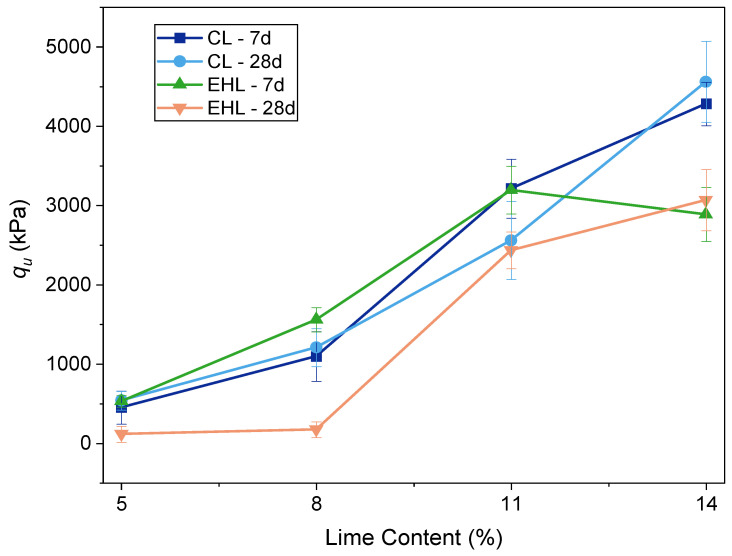
Effect of lime content and curing time on q_u_ for CL- and EHL-treated specimens.

Figure 8 shows the variation in q_u_ as a function of lime content for the two lime sources evaluated, CL and EHL (without NaOH), at 7 and 28 days of curing. The error bars represent the standard deviation of the three tested specimens for each mixture. In general, the q_u_ increased with lime content, although the magnitude of this improvement depended on the type of lime and curing time. For CL-treated specimens, the highest q_u_ values were obtained at 14% lime content, reaching 4282.63 kPa at 7 days and 4561.72 kPa at 28 days. This behavior suggests progressive strength gain with increasing lime content.

For EHL-treated specimens, the response was more variable. At 7 days, the q_u_ increased from 529.31 kPa at 5% EHL to 3195.13 kPa at 11% EHL, then decreased at 14% EHL. At 28 days, the mixtures with low EHL contents (5% and 8%) showed considerably lower strengths, whereas the highest q_u_ was obtained with 14% EHL, reaching 3070.02 kPa. This indicates that the mechanical response of EHL-treated mixtures was strongly dependent on both lime content and curing time.

The reduction in q_u_ observed for some EHL-stabilized mixtures after 28 days should be interpreted as a consequence of the particular reactivity and microstructural evolution of the eggshell-derived hydrated lime system [23]. Unlike commercial lime, EHL may exhibit a more heterogeneous particle distribution and partially carbonated or less reactive calcium-bearing phases, which can promote rapid early-age bonding but do not necessarily lead to continuous long-term cementation. At 7 days, the available Ca(OH)_2_ supplied by EHL may have favored early flocculation, particle aggregation, and localized formation of cementitious products. However, with prolonged curing, some of the calcium may have been consumed or converted into less reactive phases, while the heterogeneous fabric and residual pore network limited the development of a dense, continuous cemented matrix. This interpretation is consistent with the SEM–EDS observations, which showed localized cementitious products, as well as persistent voids and partially compacted zones in some EHL-treated specimens. Therefore, the slight reduction in strength at 28 days does not indicate the inefficiency of EHL, but rather suggests that its stabilization mechanism was more sensitive to dosage, curing time, and microstructural homogeneity than that of commercial lime.

When both stabilizers were compared, EHL showed performance similar to CL at 11% lime content after 7 days of curing. However, at 14% lime content, CL produced higher q_u_ values than EHL for both curing periods. These results suggest that although EHL can contribute to strength development, especially at intermediate and high contents, its behavior is less uniform than that of CL. Therefore, the effectiveness of EHL as an alternative stabilizer should be interpreted in light of the combined effects of dosage and curing time.

### 3.2. Influence of NaOH as an Alkaline Activator in DWTS–EHL Mixtures

**Figure 9:**
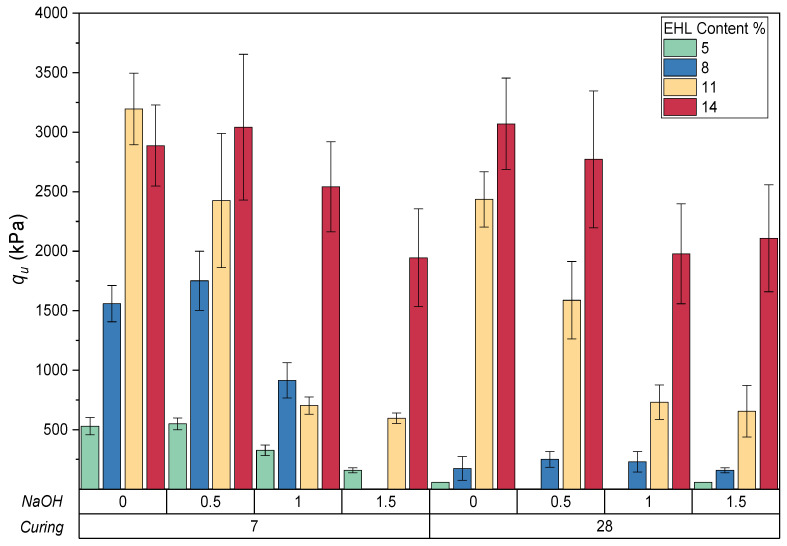
The q_u_ of soil stabilized with EHL and NaOH at 7 and 28 days of curing.

Figure 9 presents the q_u_ results of the samples stabilized with EHL, with and without alkaline activator, after 7 and 28 days of curing.

During the 7 days of curing, the incorporation of NaOH generally reduced q_u_ compared to mixtures without an alkaline activator. For the samples containing 11% EHL, the addition of 0.5, 1.0, and 1.5 M NaOH reduced the compressive strength by approximately 24%, 78%, and 81%, respectively, relative to the mixture without NaOH. Similarly, for 8% EHL, reductions of about 10%, 41%, and 62% were observed with 0.5, 1.0, and 1.5 M NaOH, respectively. The negative effect of the activator was also observed in the mixtures containing 14% EHL, where 1.0 and 1.5 M NaOH decreased the q_u_ by approximately 12% and 33%, respectively. However, the mixture with 14% EHL and 0.5 M NaOH was the only condition in which alkaline activation was beneficial, slightly increasing the strength by nearly 5% compared to the non-activated sample. Overall, the results indicate that increasing NaOH molarity tended to negatively affect early strength development.

After 28 days of curing, the adverse effect of NaOH became even more evident. For the mixtures containing 11% EHL, the presence of 0.5, 1.0, and 1.5 M NaOH decreased the q_u_ by approximately 35%, 70%, and 73%, respectively, compared to the sample without activator. Likewise, for 14% EHL, the strength reductions were approximately 10%, 36%, and 31% for 0.5, 1.0, and 1.5 M NaOH, respectively. These results indicate that alkaline activation did not promote the long-term strength development of EHL-stabilized samples, and that the detrimental effect increased with increasing NaOH molarity.

The stabilized material (DWTS) is not a conventional mineral soil but rather a sludge from a drinking water treatment plant with a particularly complex geochemical and mineralogical profile, as described by the XRF and XRD analyses. It contains 40.9% SiO_2_, 19.9% Al_2_O_3_, 4.1% Fe_2_O_3_, and just 2.0% CaO; furthermore, its solid fraction is dominated by an amorphous phase, quartz, muscovite, kaolinite, and illite, with irregular, porous, and microcracked particles. In this context, commercial lime achieved the best mechanical performance, with a q_u_ of 4561.72 kPa at 14% and 28 days, while eggshell-derived lime reached 3195.13 kPa at 11% and 7 days, and 3070.02 kPa at 14% and 28 days; Meanwhile, the alkaline activator, i.e., NaOH, reduced the strength parameter as its molarity increased, indicating in this particular case that the best results were obtained from the EHL without NaOH.

This result contrasts with a significant portion of the recent literature on eggshells, where the response is typically positive when available calcium is combined with a silica or alumina source that is more reactive than that present in DWTS. Shaji and Divya [35] showed that 3% eggshell lime reduced the liquid limit from 74% to 53%, increased the plastic limit from 27% to 46%, and raised the q_u_ to 757 kPa at 28 days; Yang et al. [23] reported that calcined eggshell in subgrade soils increased the q_u_ by up to 1000% and the CBR by up to 420%, with optimal levels of 6–8%; and Lu et al. [13] showed that a mixture of 9% eggshell ash and 8% silica fume increased the unconfined compressive strength to 1.90 MPa at 28 days. In parallel, the review by Hasheminezhad et al. [36] summarizes that eggshell powder contents of up to about 10% are generally effective but emphasizes that the response depends heavily on the clay mineralogy, shell processing, and curing [35].

The discrepancy between this study and the literature does not suggest that eggshells are an ineffective stabilizer per se; rather, it suggests that the NaOH was chemically mismatched with the DWTS matrix. The key factor is the combination of high silica–alumina content, low free calcium, moderate soluble salts, organic matter, and a porous microstructure. With only 2.0% CaO in the slurry, the pH increase caused by NaOH may have favored the surface dissolution of aluminosilicate phases, but without sufficient Ca^2+^ available to predominantly precipitate densifying C–S–H or C–A–S–H phases. In matrices with low calcium content, sodium can shift from acting as an activator to primarily functioning as a dispersing cation: it increases the system’s sodicity, expands the diffuse double layer, and weakens particle-particle contact. This interpretation is consistent with Luo et al. [37], who identified the percentage of exchangeable sodium as the most influential variable on clay dispersion; with Du et al. [38], who showed that NaOH in kaolinitic systems intensifies electrostatic repulsion forces; and with Lu et al. [39], who reviewed how alkaline solutions degrade the hydro-mechanical properties of compacted bentonites. Based on the above, it is possible to conclude that the slurry stabilized with EHL and NaOH did not encounter a matrix ready for geopolymerization, but rather a matrix that could disperse before cementing [37].

An additional explanation for the limited effectiveness of NaOH may be related to the mineralogical composition of the DWTS. Although the material contains significant amounts of SiO_2_ and Al_2_O_3_, the XRD analysis revealed that a considerable fraction of the silica is associated with quartz and other crystalline phases, which exhibit low reactivity under ambient alkaline activation conditions. Consequently, the availability of soluble silica may have been insufficient to sustain extensive geopolymeric or pozzolanic reactions capable of generating large quantities of C–S–H, C–A–S–H, or related binding phases. This interpretation may explain why increasing NaOH concentration did not improve strength development and, in some cases, produced adverse effects [40,41].

### 3.3. Failure Pattern

**Figure 10:**
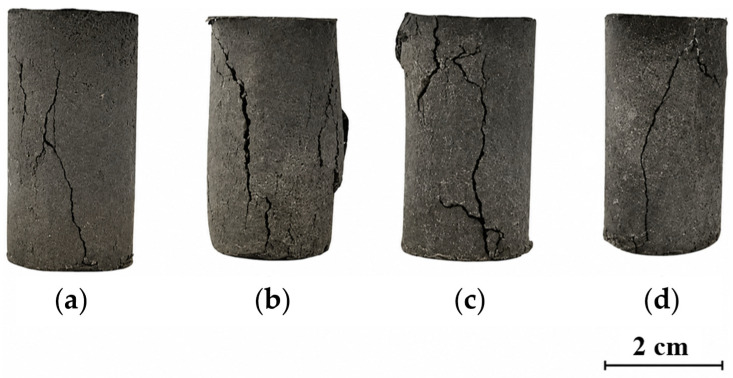
Failure patterns and deformations of specimens stabilized with commercial lime at 28 days. (**a**) 5% lime. (**b**) 8% lime. (**c**) 11% lime. (**d**) 14% lime.

During the unconfined compression tests performed on specimens stabilized with different types of lime (commercial lime and eggshell lime), several failure and deformation patterns were identified, as illustrated in Figure 10a–d. The following failure modes and deformations were observed in some of the tested specimens.

For the specimens stabilized with commercial lime (Figure 10), increasing the lime content altered the failure behavior. In sections (a) and (b), failure occurred mainly through intermittent longitudinal cracks, indicating a brittle behavior with limited stress redistribution capacity. As the lime dosage increased in sections (c) and (d), the response became more stable, exhibiting diagonal, inclined, and continuous cracks, which are typical of a material with higher consolidation and strength. This behavior is consistent with the mechanical results obtained.

**Figure 11:**
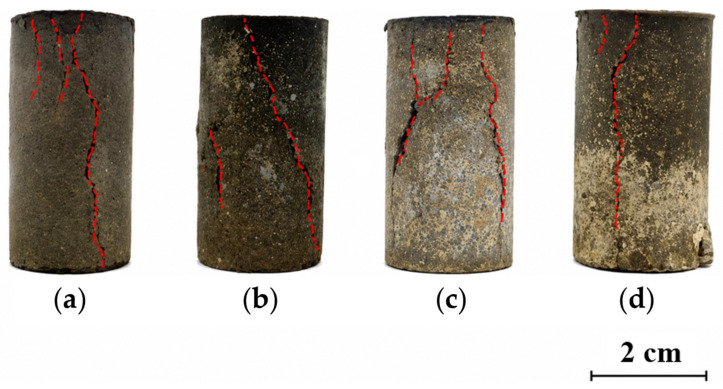
Failure patterns and deformations of specimens stabilized with EHL with 0 M NaOH at 28 days. (**a**) 5% lime. (**b**) 8% lime. (**c**) 11% lime. (**d**) 14% lime.

In the specimens stabilized with EHL without NaOH addition (Figure 11), the failure modes also showed differences associated with the lime content. For the lower contents (sections (a) and (b)), vertical fissures and partial cracks were observed, whereas at intermediate and higher dosages (sections (c) and (d)), failure occurred through more extensive inclined cracks, suggesting greater material stiffness, although without reaching the performance achieved with commercial lime.

### 3.4. Statistical Analysis of DWTS Stabilized with Alkaline-Activated EHL After 7 and 28 Days of Curing

Table 4 presents the results of the multifactorial ANOVA, which show that q_u_ was significantly influenced by NaOH concentration, EHL content, and curing time.

Likewise, the interactions among the factors were statistically significant, including the triple interaction involving NaOH, % EHL, and curing time. This indicates that the effect of each variable does not act independently but rather depends on the specific combination of the other factors. In this regard, the mechanical response of the material cannot be explained solely by changes in molarity, EHL content, or curing time; these parameters interact during the stabilization process.

Lime content was the dominant factor in the increase in strength, with higher average values observed at 11% and 14% lime content. The best responses were mainly obtained with low molarities, particularly 0 and 0.5 M, whereas increasing the molarity to 1 and 1.5 M tended to reduce the strength. The most favorable combination was 0 M, 11% lime, and 7 days of curing, with an average strength of 3195 kPa.

### 3.5. SEM-EDS Microanalysis

**Figure 12:**
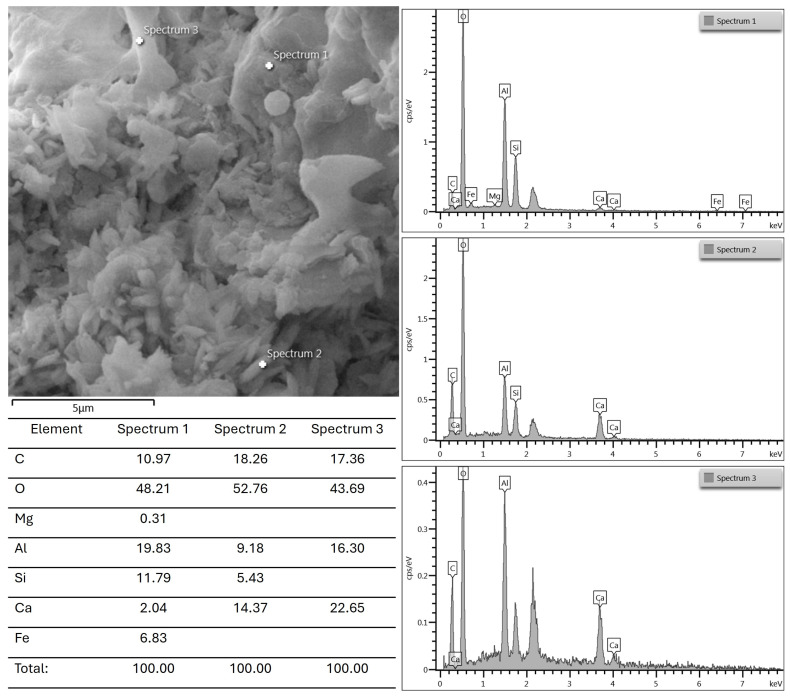
SEM-EDS of DWTS stabilized with EHL and 28 days of curing.

Figure 12 presents the microstructure of the sample stabilized with EHL (8%) after 28 days of curing. A heterogeneous matrix composed of irregular particles and partially compacted zones is observed, with no clear acicular morphologies.

The EDS analysis showed a predominance of O, Al, and Si, elements associated with the aluminosilicate phases of the sludge, together with localized increases in Ca attributed to the incorporation of eggshell lime. In some spectra, Ca contents up to 22.65% were observed, indicating partial interaction between the two materials.

The limited development of secondary cementitious products and the persistence of internal voids suggest that the calcium supplied by EHL was insufficient to generate a dense and continuous cemented matrix, which explains the lower q_u_ values compared to the CL-treated mixtures, associated with the reaction between the calcium provided by the EHL and the reactive phases present in the DWTS.

**Figure 13:**
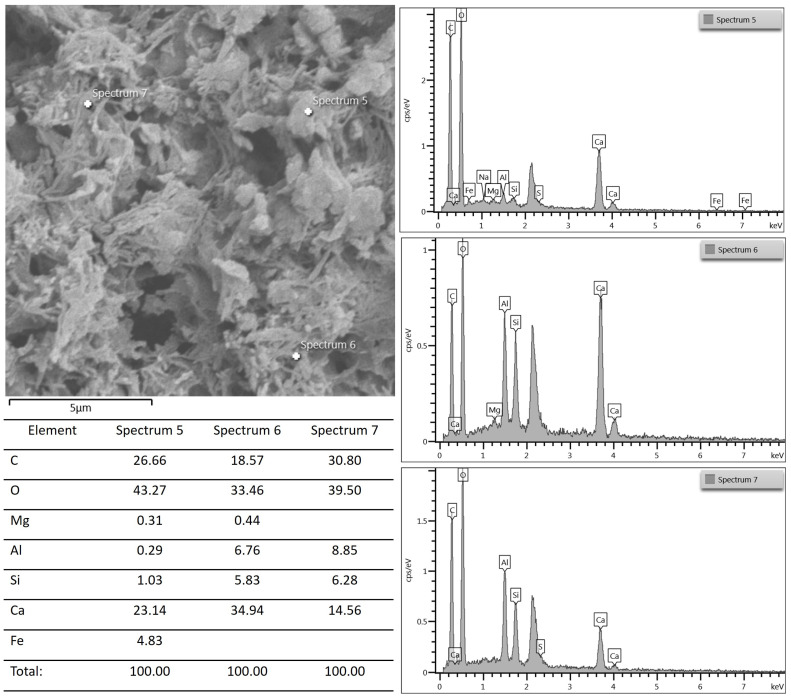
SEM-EDS DWTS stabilized with EHL, 28 days of curing, and 0.5%NaOH.

Figure 13 (DWTS mixed with 8%EHL and 0.5%NaOH cured before 28 days) reveals a heterogeneous microstructure composed of irregular agglomerates, partially compacted regions, and visible internal voids. Fibrous and laminar formations are distributed throughout the matrix, although no well-defined acicular morphologies were identified in the analyzed area.

EDS analysis confirmed the predominance of calcium in the selected points, with contents ranging from 14.56 wt% to 34.94 wt%. Aluminum and silicon were also detected, reflecting the aluminosilicate composition of the DWTS and indicating that some interaction occurred between the sludge minerals and the calcium introduced through the EHL.

Sulfur was not identified in the spectra. This indicates that the formation of AFt phases, such as ettringite, was not evident in this sample. Instead, the microstructure appears to be associated with localized calcium-rich reaction products and partially developed cementitious compounds distributed within the matrix.

**Figure 14:**
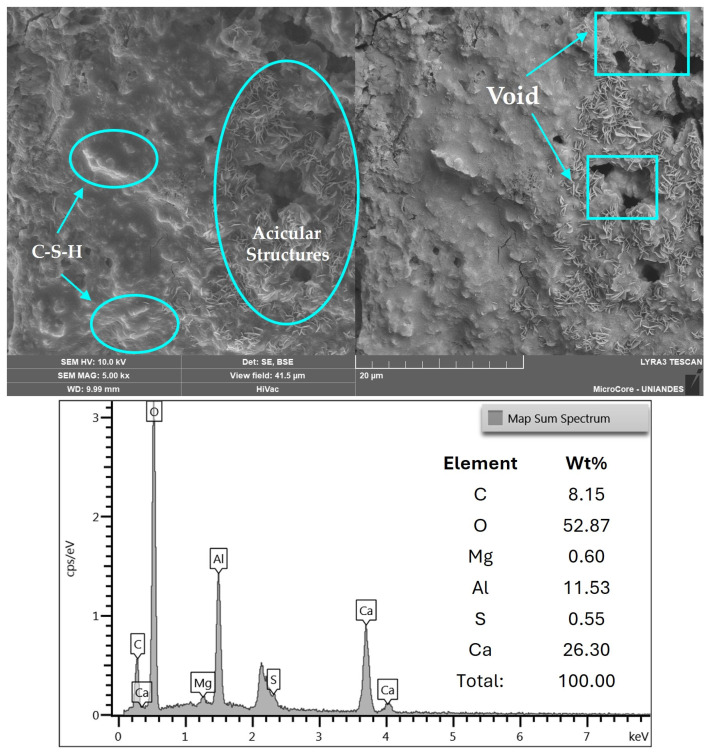
SEM and EDS of DWTS stabilized whit 11% EHL after 28 days of curing.

Figure 14 shows soil stabilization with 11% EHL. The SEM micrograph reveals acicular structures distributed within a more compact matrix, mainly on the right side of the image, with some voids. The EDS analysis showed a predominant presence of Ca, Al, and O, with minor amounts of S. The following values were recorded: Ca = 23.59%, Al = 8.84%, and S = 2.63%. The combination of Ca–Al–S–O and the observed structures suggest the possible formation of hydrated calcium sulfoaluminate phases compatible with ettringite.

Mineralogy confirms this interpretation. Quartz and muscovite are much less reactive at room temperature than highly reactive amorphous ash or slag; kaolinite and illite can indeed participate, but not at the same rate or to the same extent as a calcined or vitrified source. In fact, Nouhi et al. [42] showed that low-plasticity clays respond better to alkaline activation than high-plasticity clays. Yet, they noted that activated soils are vulnerable to cracking and expansion due to alkali-silica reactions. DWTS, in addition to being amorphous, contains quartz and lamellar clays and exhibits extremely high plasticity; this combination increases the likelihood of heterogeneous response, discontinuous gel formation, and microdamage. Therefore, although the composition of DWTS contains silica (40.9%) and alumina (19.9%), the absence of a robust calcium source within the sludge and the lack of a highly reactive complementary precursor make it plausible that the net effect of the NaOH was dispersion rather than cementation.

Organic matter and fine-grained particles also contribute to this trend. In mixtures with WTS or DWTS, several authors have shown that an increase in fines can reduce strength or require a different stabilizer: Takao et al. [43] observed that, in a sandy loam soil, an increase in WTS reduced the CBR in systems with cement but increased it in systems with lime, precisely because the lime interacted better with the fine particles; Kafle and Baghbani [8] reported that WTS alone tended to initially decrease the CBR due to its high content of fines and organic matter; and Boscov et al. [6] highlight in their review that the geotechnical performance of sludge depends on carefully controlling moisture, plasticity, and internal structure. In the present case, the electrical conductivity (EC) of 2.65 dS/m, the organic carbon content of 1.91%, the microcracked morphology, and the high molding water content make it reasonable to conclude that NaOH increased the system’s susceptibility to dispersion, local swelling, and heterogeneity, rather than producing a dense and continuous matrix [43].

This mechanism also explains why NaOH is effective with other soil-binder combinations but not with DWTS-EHL. Pourakbar et al. [44] increased the q_u_ to 813 kPa at 7 days and 1.46 MPa at 56 days with 2.5% activated sludge treated with NaOH and sodium silicate. Bijalwan et al. [45] achieved 2250 kPa and a CBR of 87% in geopolymers with WTS+FA+GGBS, where NaOH acted in conjunction with a clearly reactive mixture and a favorable SS/SH ratio; meanwhile, Isik [46] reported increases of 8.1, 12.2, and 14. 6-fold increases in the q_u_ of a clay stabilized with a construction waste geopolymer, but under a different precursor-dose-curing system; and Tanyıldızı [21] showed that alkaline-activated corn stover ash enhanced with eggshell powder produced more resistant subgrades with lower equivalent CO_2_ emissions than OPC. The constant in these studies is not NaOH, but rather: NaOH + suitable precursor + sufficient reactive silica/alumina + available calcium + compatible curing structure [47,48].

A comparative analysis of the microstructural observations indicates that both commercial lime (CL) and eggshell-derived hydrated lime (EHL) promoted the formation of cementitious products responsible for strength development in the stabilized DWTS. However, important differences were observed between the two stabilizers. Specimens treated with CL generally exhibited a denser, more homogeneous matrix, characterized by a lower visible void ratio and greater particle bonding. These features are consistent with the higher unconfined compressive strengths obtained for CL-stabilized mixtures (up to 4.56 MPa after 28 days of curing).

The EHL-stabilized specimens also showed the formation of reaction products compatible with C-A-S-H gels and calcium sulfoaluminate hydrates, including possible ettringite structures. Nevertheless, the micrographs revealed a slightly more porous fabric and a less continuous cemented matrix compared with CL-treated samples. This observation agrees with the lower, although still significant, compressive strengths achieved by EHL-stabilized DWTS (up to approximately 3.1 MPa). Therefore, the SEM-EDS results support the mechanical findings and demonstrate that while CL generated a more effective cementation network, EHL was capable of producing similar stabilization mechanisms and represents a technically viable and sustainable alternative for DWTS stabilization.

## 4. Engineering Applications of Improved DWTS

Based on the obtained compressive strength values, the stabilized DWTS mixtures may be classified according to their potential engineering application. Mixtures containing 5–8% EHL exhibited moderate strength development and may be suitable for improved fills and low-load earthworks. Conversely, mixtures containing 11–14% EHL achieved strengths exceeding 3 MPa, indicating their potential use as compacted structural fills, retaining wall backfills, and engineered embankments. The maximum strength was observed in the mixture containing 14% CL (4.56 MPa), suggesting its applicability to projects requiring enhanced mechanical performance.

These values are within or above the ranges commonly reported for stabilized structural fills, improved subgrades, and engineered embankments. Consequently, the stabilized DWTS mixtures may be considered potential candidates for compacted fills behind retaining walls, pavement subgrade improvement, working platforms, and low-rise earth structures.

**Figure 15:**
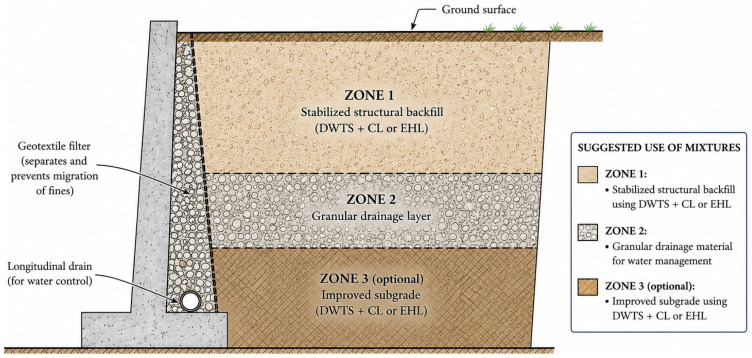
Proposed application of stabilized DWTS mixtures as engineered backfill in retaining wall systems.

Figure 15 illustrates a potential application of the stabilized DWTS mixtures as compacted backfill behind retaining walls. The ASTM D4609 [49] establishes that stabilized soils should exhibit sufficient mechanical improvement and durability for engineering use. The mixtures evaluated in this study reached q_u_ values of approximately 3.0–4.5 MPa, exceeding the typical strength ranges adopted for stabilized structural fills, suggesting their potential applicability in retaining wall backfill systems when combined with adequate drainage and compaction control. However, field implementation would require additional evaluation of hydraulic conductivity, durability under wet–dry cycles, and long-term deformation characteristics.

From the perspective of eggshell-derived lime, this study and recent evidence remain favorable. Saldanha et al. [50] showed that eggshell-derived limes have a composition suitable for stabilization, with up to 97.0% CaO for quicklime and 89.6% Ca(OH)_2_ for hydrated lime, and reduce damage to ecosystem quality by 65.1% and 50%, respectively, compared to their limestone-derived equivalents. Nierwinski et al. [16] reinforced this line of research, showing that eggshell-derived hydrated lime outperformed commercial hydrated lime in a CH clay with fly ash, and attributed this advantage to smaller particle size and higher calcium purity. Consequently, the performance of this derivative depends on the dominant chemical pathway being calcium-pozzolanic rather than sodium-dispersive. Thus, the contribution of EHL was competitive but insufficient to outperform commercial lime, and activation with NaOH exacerbated this imbalance.

Beyond the mechanical improvements enabled by stabilization, the proposed approach offers significant environmental and economic benefits. Both DWTS and eggshell waste are commonly disposed of in landfills, generating environmental burdens and disposal costs. Their reuse as components of geotechnical materials promotes circular economy principles by converting waste streams into value-added products. Furthermore, the use of eggshell-derived hydrated lime (EHL) as an alternative calcium source may reduce demand for conventional lime production, thereby lowering consumption of virgin raw materials and potentially reducing the carbon footprint associated with waste management and construction activities. Therefore, the proposed stabilization strategy simultaneously addresses waste valorization, resource efficiency, and sustainable infrastructure development.

## 5. Conclusions

The present study demonstrates that the DWTS obtained from the drinking water treatment plant of Cartagena de Indias can be stabilized and transformed into a dense and mechanically resistant material. In addition to commercial lime, eggshell-derived hydrated lime (EHL) demonstrated favorable results:

− Commercial lime exhibited the best overall mechanical performance, reaching a maximum unconfined compressive strength of 4561.72 kPa with 14% addition after 28 days of curing. In contrast, EHL showed a competitive, although less uniform, behavior. The highest q_u_ obtained with this material was 3195.13 kPa with 11% EHL after 7 days, while at 28 days the maximum value reached 3070.02 kPa with 14% EHL. These results indicate that eggshell waste can serve as an alternative calcium source for DWTS stabilization, particularly at medium and high levels.
− Alkaline activation with NaOH did not improve the performance of the DWTS–EHL system. Increasing NaOH molarity progressively reduced the q_u_, particularly at 1.0 and 1.5 M, suggesting that, under the evaluated conditions, NaOH does not act as an efficient activator for this type of sludge. This behavior may be attributed to the low CaO content, the high aluminosilicate fraction, the elevated plasticity, and the presence of microcracks, organic matter, and salts, all of which limit the formation of a continuous cementitious matrix.
− The most suitable stabilization route for this DWTS is calcium-based rather than sodium-alkaline. Commercial lime at 14% provided the highest mechanical strength, while EHL without NaOH emerges as a technically and environmentally viable alternative. Nevertheless, life cycle assessment and sustainability analyses are recommended to comprehensively validate the reuse of both DWTS and EHL in stabilization applications.
− This study was limited to a single DWTS source and short-term curing periods. Additional studies evaluating durability, permeability, cyclic wet–dry performance, and field-scale applicability are required.

## Figures and Tables

**Table 1:** Properties of the soil sample.

Property of DWTS	Standard/Reference	Value	Unit
Consistency limits			
Plasticity limit, P.L.	[24]	161.60	%
Plastic index, P.I.	[24]	149.67	%
Specific gravity, Gs	[25]	2.67	-
pH in saturation paste	-	6.86	-
Electrical conductivity	-	2.65	dS/m
Moisture retention	-	10.1	%
Cation exchange capacity	-	8.40	Meq/100 g
Total oxidizable organic carbon	-	1.91	%
Optimum moisture content	-	29.3	%
Maximum dry unit weight	-	1.66	g/cm^3^
Density, dry basis	-	0.996	g/cm^3^
Loss on ignition (LOI)	-	6.30	%

**Table 2:** Chemical composition of the DWTS sample determined by XRF: major oxides (% *w*/*w*) and detected trace elements (ppm).

Major Oxides	Content (% *w*/*w*)	Trace Elements	Content (ppm)
SiO_2_	40.9	Rb	1228
Al_2_O_3_	19.9	Bi	783
Fe_2_O_3_	4.1	V	238
TiO_2_	1.3	Th	224
CaO	2.0	Pb	158
K_2_O	1.9	Zn	137
P_2_O_5_	<1.0	Sr	65
		Zr	62
		Mo	38

**Table 3:** Experimental matrix for DWTS specimens stabilized with CL and EHL.

Binder System	Binder Content (%)	NaOH (M)	Dry Unit Weight (kN/m^3^)	Moisture Content (%)	Curing Time (Days)	Number of Specimens
DWTS-CL	5, 8, 11, 14	—	15.6	29.3	7 and 28	24
DWTS-EHL	5, 8, 11, 14	0	15.6	29.3	7 and 28	24
DWTS-AA-EHL	5, 8, 11, 14	0.5	15.6	29.3	7 and 28	24
DWTS-AA-EHL	5, 8, 11, 14	1.0	15.6	29.3	7 and 28	24
DWTS-AA-EHL	5, 8, 11, 14	1.5	15.6	29.3	7 and 28	24

**Table 4:** ANOVA table for the results.

Source	Sum of Squares	Degrees of Freedom	Mean Squares	Z	*p*-Value	Significance (*p*-Value < 0.05)
Molarity (NaOH)	1.98071 × 10^7^	3	6,602,381.7564	63.55012	<0.0001	yes
%EHL	7.27827 × 10^7^	3	2.42609 × 10^7^	233.51922	<0.0001	yes
Curing time (t)	5,282,769.7501	1	5,282,769.7501	50.84842	<0.0001	yes
NaOH × %EHL	9,738,402.94338	9	1,082,044.77149	10.41504	<0.0001	yes
NaOH × t	1,788,908.07135	3	596,302.69045	5.73961	0.00153	Yes
%EHL × t	1,382,124.42908	3	460,708.14303	4.43447	0.00679	Yes
NaOH × %EHL × t	2,550,459.91674	9	283,384.43519	2.72767	0.0093	yes
Model	1.13333 × 10^8^	31	3,655,887.2962	35.18913	<0.0001	yes
Error	6,649,120.74193	64	103,892.51159			
Corrected Total	1.19982 × 10^8^	95				

*R*^2^ = 0.945.

## Data Availability

The original contributions presented in this study are included in the article. Further inquiries can be directed to the corresponding authors.

## Abbreviations

The following abbreviations are used in this manuscript:

AA	Alkaline activator
AFt	Alumina–ferric oxide–trisulfate phase
ANOVA	Analysis of variance
ASTM	American Society for Testing and Materials
BSE	Backscattered electrons
C–A–H	Calcium aluminate hydrate
C–A–S–H	Calcium aluminosilicate hydrate
C–S–H	Calcium silicate hydrate
CBR	California Bearing Ratio
CEC	Cation exchange capacity
CL	Commercial lime
DWTS	Drinking water treatment sludge
EDS	Energy-dispersive X-ray spectroscopy
EHL	Eggshell-derived hydrated lime
ESP	Eggshell powder
FA	Fly ash
GGBS	Ground granulated blast-furnace slag
Gs	Specific gravity
LOI	Loss on ignition
NaOH	Sodium hydroxide
OPC	Ordinary Portland cement
P.I.	Plasticity index
P.L.	Plastic limit
ppm	Parts per million
q_u_	Unconfined compressive strength
R^2^	Coefficient of determination
SE	Secondary electrons
SEM	Scanning electron microscopy
SEM–FIB	Scanning electron microscopy–focused ion beam
SPSS	Statistical Package for the Social Sciences
SS/SH	Sodium silicate/sodium hydroxide ratio
WDS-XRF	Wavelength-dispersive X-ray fluorescence
wt.%	Weight percent
WTS	Water treatment sludge
XRD	X-ray diffraction
XRF	X-ray fluorescence
